# Novel Insights into the Circadian Rhythms Based on Long Noncoding and Circular RNA Profiling

**DOI:** 10.3390/ijms25021161

**Published:** 2024-01-18

**Authors:** Xiaodong Tan, Jiawen Zhang, Jie Dong, Minjie Huang, Zhenzhen Zhou, Deqian Wang

**Affiliations:** Institute of Animal Husbandry and Veterinary Science, Zhejiang Academy of Agricultural Sciences, Hangzhou 310021, China; tanxiaodong08@163.com (X.T.);

**Keywords:** circadian rhythm, long noncoding RNA, circular RNA, hypothalamus, *AOX1*

## Abstract

Circadian rhythm disorders pose major risks to human health and animal production activity, and the hypothalamus is the center of circadian rhythm regulation. However, the epigenetic regulation of circadian rhythm based on farm animal models has been poorly investigated. We collected chicken hypothalamus samples at seven time points in one light/dark cycle and performed long noncoding RNA (lncRNA), circular RNA (circRNA), and mRNA sequencing to detect biomarkers associated with circadian rhythm. We enhanced the comprehensive expression profiling of ncRNAs and mRNAs in the hypothalamus and found two gene sets (circadian rhythm and retinal metabolism) associated with the light/dark cycle. Noncoding RNA networks with circadian expression patterns were identified by differential expression and circadian analysis was provided that included 38 lncRNAs, 15 circRNAs, and 200 candidate genes. Three lncRNAs (ENSGALT00000098661, ENSGALT00000100816, and MSTRG.16980.1) and one circRNA (novel_circ_010168) in the ncRNA–mRNA regulatory network were identified as key molecules influencing circadian rhythm by regulating *AOX1* in retinal metabolism. These ncRNAs were predicted to be related to pernicious anemia, gonadal, eye disease and other disorders in humans. Together, the findings of this study provide insights into the epigenetic mechanisms of circadian rhythm and reveal *AOX1* as a promising target of circadian rhythm regulation.

## 1. Introduction

Circadian rhythm describes the fluctuations of various physiological processes and behaviors in vivo within 24 h, in synchrony with the daily light/dark cycle [1]; almost every species exhibits some circadian rhythms. Circadian rhythm dysfunction can influence fundamental metabolism and vital activities, causing obesity, depression, and stunted growth, among other disorders. Circadian rhythm is driven by the circadian timing system consisting of a central molecular clock (located in the suprachiasmatic nucleus (SCN) of the hypothalamus), which is the regulatory center of the circadian rhythm, and peripheral clocks (present in peripheral tissues, e.g., the liver, heart, or spleen) in mammals [2]. The SCN orchestrates the rhythm in the peripheral tissues by broadly regulating gene expression. Two feedback loops involving clock genes constitute the central molecular clock. The positive genes *BMAL1* and *CLOCK* have an initiating effect on the negative factors CRY (*CRY1* and *CRY2*) and PER (*PER1*, *PER2*, and *PER3*) in the first loop and on *NR1D1* and *NR1D2* in the second loop. Both of the negative genes in the two loops could in turn repress the transcription of *BMAL1* and *CLOCK*, resulting in the start of the next cycle.

Circadian rhythm is maintained by zeitgeber, which is the name given to external environmental signals that restrain the internal timing system, such as light [3]. The SCN, acting as the regulatory center of circadian rhythm, receives the light/dark signals from the retina and transmits them to other regions in the brain and peripheral tissues [4]. Therefore, to dissect the molecular mechanism of circadian rhythm, it is a top priority to investigate the regulatory network in SCN during the light/dark cycle. In sheep, one novel lncRNA, XR_173257.3, and the target gene *AANAT* were shown to be important for circadian rhythm and seasonal reproduction [5]. Jiang et al. found that exposure to green light increased the expression of the *CLOCK*, *BMAL1*, and *CRY1* genes in the hypothalamus of chicks at six time points [6]. An intermittent photoperiod enhanced the transcription of *BMAL1*, *BMAL2*, *CRY1*, and *CRY2* in the hypothalamus and altered the expression of certain circadian genes in the liver (*CLOCK*, *BMAL1*, and *CRY2*) and cecal wall (e.g., *BMAL1*, *CRY1*, and *CRY2*) [7]. Recent investigations of the mechanisms regulating circadian rhythm have focused on the genome-wide transcription and translation of protein-coding genes such as PER, BMAL, and CRY in SCN or pineal gland tissue [8], but it is believed that additional genes and posttranscriptional regulators (such as noncoding RNAs) may perform crucial roles in the maintenance of circadian rhythms and may be promising markers.

Noncoding RNAs (ncRNAs) are important epigenetic regulators and influence gene transcription by physically interacting with DNA, mRNA, and miRNA, and protein through structural domains generated by RNA folding [9]. LncRNAs are characterized by relatively low expression levels, tissue specificity, and weak conservation and play important roles in biological processes, e.g., circadian rhythms. Although most past research related to circadian rhythms has focused on the mRNA markers, the functions of lncRNAs have been detected in some animals. Zhang et al. reported that more than 1000 lncRNAs are rhythmically expressed in different tissues in mice [10]. Over 700 circadian lncRNAs in the pineal gland and testis were found in zebrafish [11]. Si et al. revealed that some lncRNAs associated with the immune response and metallopeptidase activity could contribute to the circadian disruptions [12]. Additionally, circular RNAs (circRNAs), a large class of highly stable RNA molecules that consist of multiple or single exons, are highly transcribed in the brain and are of great importance in circadian rhythm [13,14]. Due to their high stability and synaptic enrichment, these circRNAs are thought to be involved in intracellular information transport, memory-related functions, and neurodevelopment [15,16]. Abnormal wake/sleep cycles can cause many disorders in parallel [17,18,19], indicating the significance of investigating the function of circRNAs in circadian rhythm. Zheng et al. reported that circ-WNK2 promotes elevated levels of the circadian gene *AANAT* by sponging miR-328a-3p in rat pineal gland tissue [20]. Ivanov et al. proved that circRNA Cdr1as is regulated during 12:12 h light/dark cycles in the SCN and affects the adaptation to daily changes [21]. Zhao et al. found that the circadian gene *BMAL1* could bind to the promoter of circGUCY1A2 and increases its expression in non-small cell lung cancer cases [22]. In total, noncoding RNAs play a broad regulatory role in circadian rhythm; however, poor understanding has restricted the discovery of powerful lncRNAs or circRNAs markers [23,24].

In this study, to detect candidate regulatory molecules affecting circadian rhythm, we investigated the transcriptional patterns of noncoding RNAs (lncRNAs and circRNAs) and mRNAs in the hypothalamus of chickens under a 12:12 h light/dark cycle. LncRNAs, circRNAs, and mRNAs with clear circadian expression patterns in the hypothalamus were identified based on the differential expression and rhythmic analysis, and the potential networks were constructed with strong correlations. Our findings revealed that the MSTRG.16890.1/novel_circ_010168-*AOX1* axis plays an important role in regulating mechanism of hypothalamic circadian rhythm through retinol metabolism.

## 2. Results

### 2.1. Circadian lncRNA/circRNA Profiling in the Hypothalamus

**Figure 1:**
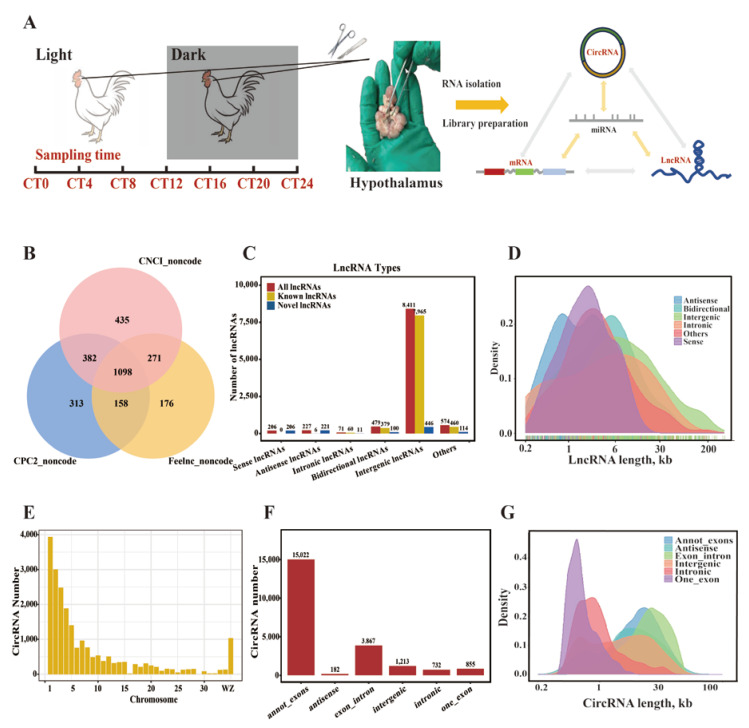
Overview of lncRNA and circRNA expression in the hypothalamus. (**A**) Hypothalamus sampling and sequencing strategy in one light/dark cycle. The mRNA, lncRNA, and circRNA of the hypothalamus were sequenced while microRNA was not sequenced. (**B**) Identification of novel lncRNAs in the hypothalamus. (**C**) Statistics for lncRNA numbers based on different types. (**D**) Distribution of length of lncRNAs. The axis of lncRNA length was adjusted in log2 scale. (**E**) Distribution of circRNA in each chromosome of chicken. (**F**) Statistics for circRNA numbers based on different types. (**G**) Distribution of length of lncRNAs. The axis of lncRNA length was adjusted in log2 scale.

To investigate the role of ncRNAs in the circadian rhythm system, lncRNA and circRNA sequencing of the hypothalamus at seven time points (CT0~CT24) was performed (Figure 1A). Over 92% of 163.5 Gb trimmed data were mapped to the chicken reference genome (GRCg6a), and 70% of mapped reads were aligned to exon regions (Table S1, Figure S1). Based on genome annotation, a total of 17,256 mRNAs (16,877 known and 379 novel mRNAs), 6705 lncRNAs (5607 known and 1098 novel lncRNAs), and 21,871 circRNAs were annotated and predicted (Table S1).

For lncRNA, we used three methods to predict the coding ability and screen the noncoding transcripts. A total of 1098 novel lncRNAs were defined as mentioned above (Figure 1B), 206 sense lncRNAs and 221 antisense lncRNAs were identified, and most lncRNAs were located in the intergenic regions (Figure 1C). In total, 4198~4351 of 6705 lncRNAs were expressed at each time point (Figure S2). To verify the characteristics of lncRNAs, we calculated and summarized transcript length and exon number (Figure 1D and Figure S3). The length of the lncRNAs was generally <10 kb (Figure 1D).

For circRNAs, 4920~7534 of 21,871 circRNAs were detected in each CT using CIRIquant v1.1 software (Figure S4). Over 58% of circRNAs were located in macrochromosomes (GGA1~GGA5) (Figure 1E), while the density showed the opposite trend; the microchromosomes (GGA30 and GGA32) covered over 10 circRNAs within the genomic region of 100 kb (Table S2). This finding is consistent with the distribution of mRNAs and lncRNAs. A total of 15,022 circRNAs were annotated from exons, and 3867 circRNAs originated from exon–intron regions (Figure 1F). A distribution analysis revealed that the length of one_exon and intronic circRNAs ranged from 200 to 2000 bp, while the length of annot_exons, antisense, and exon_intron circRNAs ranged from 3 to 10 kb (Figure 1G).

### 2.2. Identification of Candidate lncRNAs Affecting Circadian Rhythm

**Figure 2:**
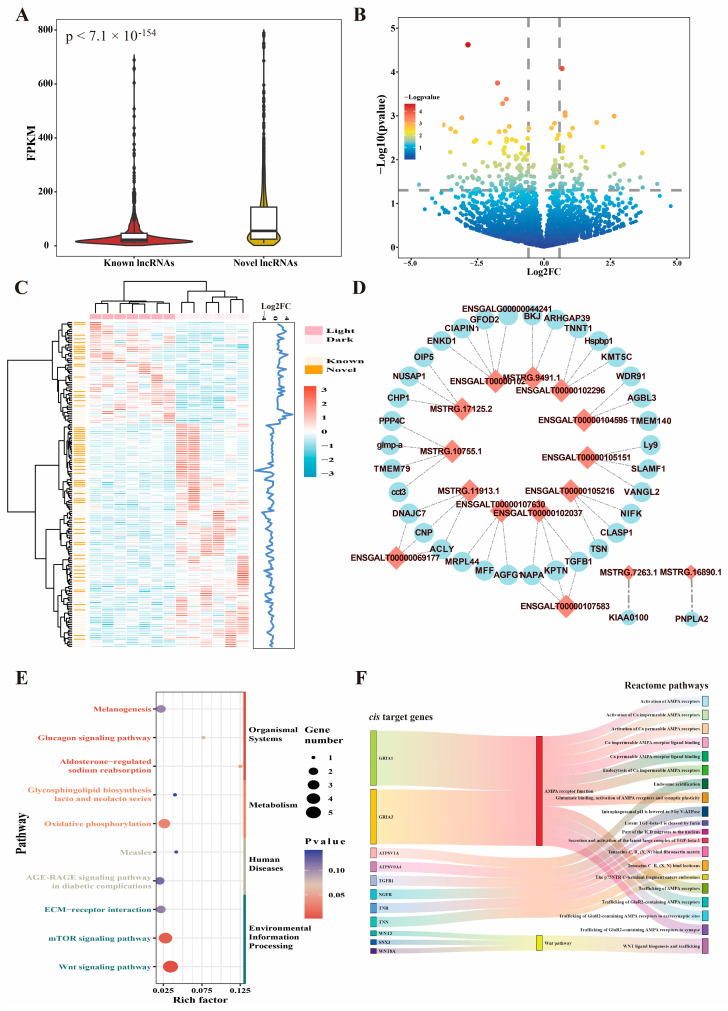
Differential expression analysis and function prediction of lncRNAs. (**A**) Comparison of expression level between known and novel lncRNAs in the hypothalamus. (**B**) Volcano plot of differentially expressed lncRNAs between the light and dark group. (**C**) Expression pattern of differentially expressed lncRNAs. The group and lncRNA type were annotated, the right part indicated the fold change of expression. (**D**) LncRNA–mRNA regulatory network based on the *cis* and *antisense* method. The light red diamond indicated the lncRNAs, while the light blue circle indicated protein-coding genes. (**E**) KEGG enrichment based on the *cis* target genes of differentially expressed lncRNAs. The enriched pathways were clustered four categories, including organismal systems, metabolism, human disease, and environmental information processing. (**F**) Reactome enrichment analysis for the *cis* target genes of differentially expressed lncRNAs.

To identify candidate lncRNAs affecting circadian rhythm, we first calculated the expression patterns and differentially expressed lncRNAs (DElncRNAs) in the light and dark groups (Figure S5). First, the expression of known and novel lncRNAs was quantified and found to be significantly higher for novel lncRNAs than for known lncRNAs using the Wilkson rank sum test (*p* < 7.1 × 10^−154^) (Figure 2A). In the hypothalamus, the sense and antisense lncRNAs were highly transcribed compared to other types of lncRNAs (*p* < 10 × 10^−49^) (Figure S6). A total of 179 lncRNAs (downregulated: 123, upregulated: 56) were differentially expressed between the light and dark groups (Figure 2B, Table S3), including 68 novel lncRNAs. The different expression patterns of DElncRNAs were obtained using the hierarchical clustering method (Figure 2C). To predict the biological functions of these DElncRNAs, a *cis* analysis was first performed and revealed 135 genes located within the 10 kb up- and down-stream of DElncRNAs (Figure S7, Table S4). Four *cis* targets were found for ENSGALT00000102331 and MSTRG.10755.1 (Figure 2D). Two target genes were potentially regulated by the novel lncRNAs (MSTRG.16890.1 and MSTRG.7263) based on *antisense* function (Figure 2D). For the putative target genes, KEGG pathway analysis revealed that four pathways (Wnt signaling pathway, mTOR signaling pathway, oxidative phosphorylation, and aldosterone-regulated sodium reabsorption) were significantly enriched (Figure 2E, Table S5). To maximize the possibility of detecting the underlying regulatory pathway, we used Reactome enrichment and found that the activity of the AMPA receptor and Wnt pathway were correlated with circadian rhythm (Figure 2F, Table S6).

### 2.3. Identification of Candidate CircRNAs Affecting Circadian Rhythm

**Figure 3:**
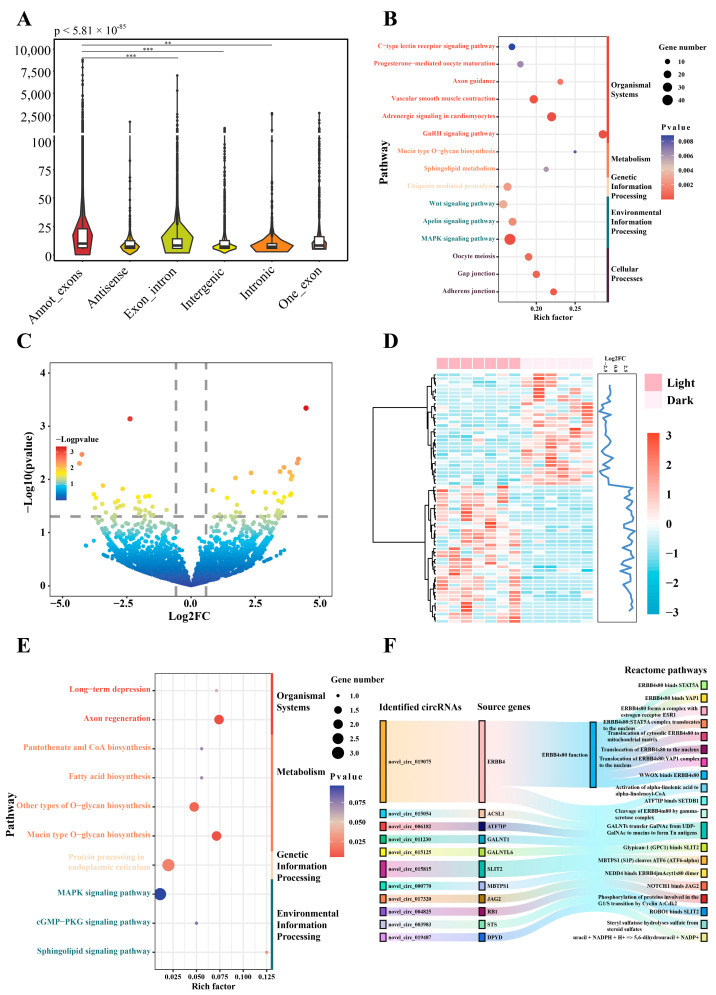
Differential expression analysis and function prediction of circRNAs. (**A**) Comparison of expression level for different type of circRNAs. ** indicated *p* < 0.01, *** indicated *p* < 0.001. (**B**) KEGG enrichment for the source genes of identified circRNAs. The enriched pathways were clustered four categories, including organismal systems, metabolism, environmental information processing, genetic information processing, and cellular processes. (**C**) Volcano plot of differentially expressed circRNAs between light and dark. (**D**) Expression pattern of differentially expressed circRNAs, the blue line in the right indicated the fold change of expression level. (**E**) KEGG enrichment analysis for differentially expressed circRNAs in the hypothalamus in the light and dark, the pathways were divided into four groups, including organismal systems, metabolism, environmental information processing, and genetic information processing. (**F**) Reactome analysis for differentially expressed circRNAs.

The expression profile of circRNAs from hypothalamus samples in light and dark was drafted in a manner similar to that used for the lncRNAs (Figure S8). The circRNAs originating from annotated exons were expressed at higher levels than other types of circRNAs (*p* < 5.81 × 10^−85^) (Figure 3A). The source genes of expressed circRNAs were annotated, and enrichment analysis showed 34 significant pathways (e.g., GnRH signaling pathway, adrenergic signaling in cardiomyocytes, and MAPK signaling pathway) (Figure 3B, Table S7). In the differential expression analysis, 69 circRNAs (downregulated: 31, upregulated: 38) were differentially expressed between light and dark conditions (Figure 3C, Table S8). Hierarchical clustering of the expression patterns indicated that the expression profiles of samples from light and dark groups segregated into distinct clusters (Figure 3D). KEGG functional analysis was conducted to explore the biological functions of these DEcircRNAs. The results showed that the dominant functions may be related to signal conduction, which was associated with five significant pathways (e.g., axon regeneration, O-glycan biosynthesis, and protein processing in the endoplasmic reticulum) (Figure 3E, Table S9). Similarly, we also performed Reactome analysis, and 101 significant reactions and functions were identified (Figure 3F, Table S10). ERBB4s80 could be a key molecule regulated by the ERBB4 gene and novel_circ_019075 in the potential mechanism of circadian rhythm.

### 2.4. Screening for Potential Gene Sets Related to Circadian Rhythm

**Figure 4:**
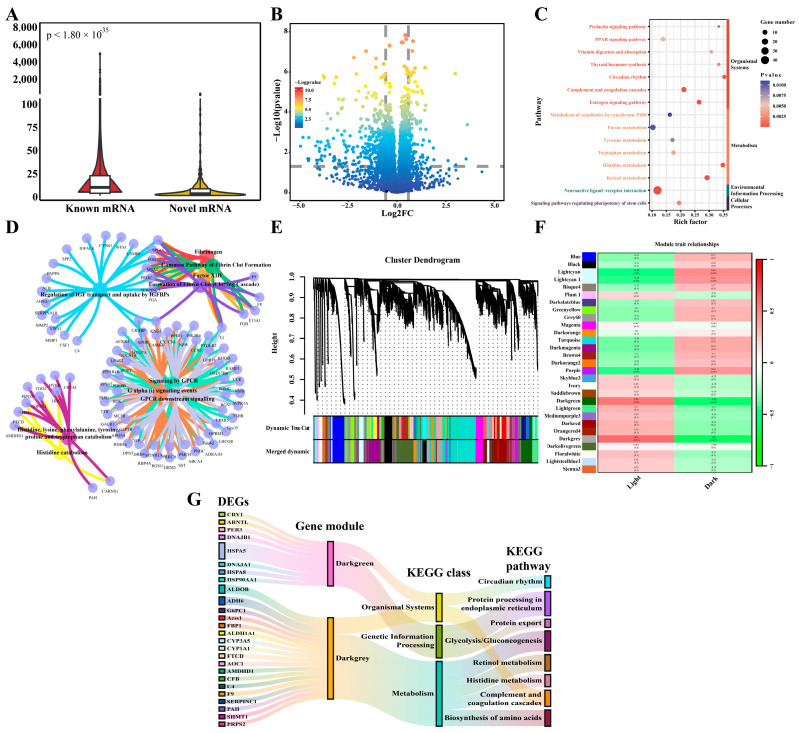
Identification of circadian genes and function analysis. (**A**) Comparison of known and novel genes in transcription level. (**B**) Volcano plot of differentially expressed genes. (**C**) KEGG enrichment for differentially expressed genes. The pathways were divided into four groups, including organismal systems, metabolism, environmental information processing, and cellular processes. (**D**) Reactome analysis for differentially expressed genes. The small circles indicate the differentially expressed genes, the bigger circles indicated the Reactome reactions. The color represents the different reactions. (**E**) Clustering and merge results for gene modules in WGCNA pipeline. (**F**) The relationship between light/dark and gene modules. (**G**) KEGG enrichment for the differentially expressed genes from two circadian gene modules.

To identify the functions of crucial mRNAs related to circadian rhythm, we quantified the sequenced mRNAs using the recommended pipeline noted above (Figure S9). The expression levels of known mRNAs were significantly higher than those of novel mRNAs (Figure 4A). Based on differential expression analysis, 826 DEGs (upregulated: 205, downregulated: 621) were found between the two groups, including 25 novel mRNAs (Figure 4B, Table S11). KEGG pathways showed that retinol metabolism, circadian rhythm, and other pathways (Figure 4C, Table S12), involving *PER3*, *CRY1*, *BMAL1*, etc., were influenced significantly. Reactome analysis revealed that the coagulation reaction and amino acid (e.g., tyrosine, tryptophan, and lysine) metabolism were important process exhibiting circadian rhythms (Figure 4D, Table S13). To infer the gene network associated with circadian rhythm, WGCNA was conducted based on the phenotypes of light and dark. The soft threshold was set to 6, according to the standard of R^2^ > 0.9, and 29 merged gene modules were obtained using a step-by-step method. Individual modules included 77~2648 genes and <190 DEGs (Figure 4E, Figures S10 and S11). According to the threshold (r > 0.5 or r < −0.5, *p* < 0.05), two candidate modules, dark grey and dark green modules, were identified as relevant to circadian rhythm (Figure 4F, Figures S12 and S13). We found that the dark green module was correlated with protein processing in the endoplasmic reticulum and circadian rhythm, among others, while the dark grey module was relevant to neuroactive ligand–receptor interactions and biosynthesis of amino acids, among others. (Figures S14 and S15, Tables S14 and S15). The dark green and dark grey modules included 79 (e.g., *CRY1*, *BMAL1*, *PER3*) and 190 (e.g., *ALDOB*, *SHMT1*, and *SERPINC1*) DEGs, respectively (Table S11). Circadian rhythm and retinol metabolism were proven to be crucial functions for the dark green and dark grey modules (Figure 4G, Tables S16 and S17). Additionally, when combining the DEGs and core genes identified by the standard of |KME| > 0.8, a total of 137 hub genes were defined, including *CRY1*, *HSPA5*, and *WNT4* (Figure S16, Table S18).

### 2.5. Circadian Expression and Trend Analysis for Candidate lncRNA/circRNA

**Figure 5:**
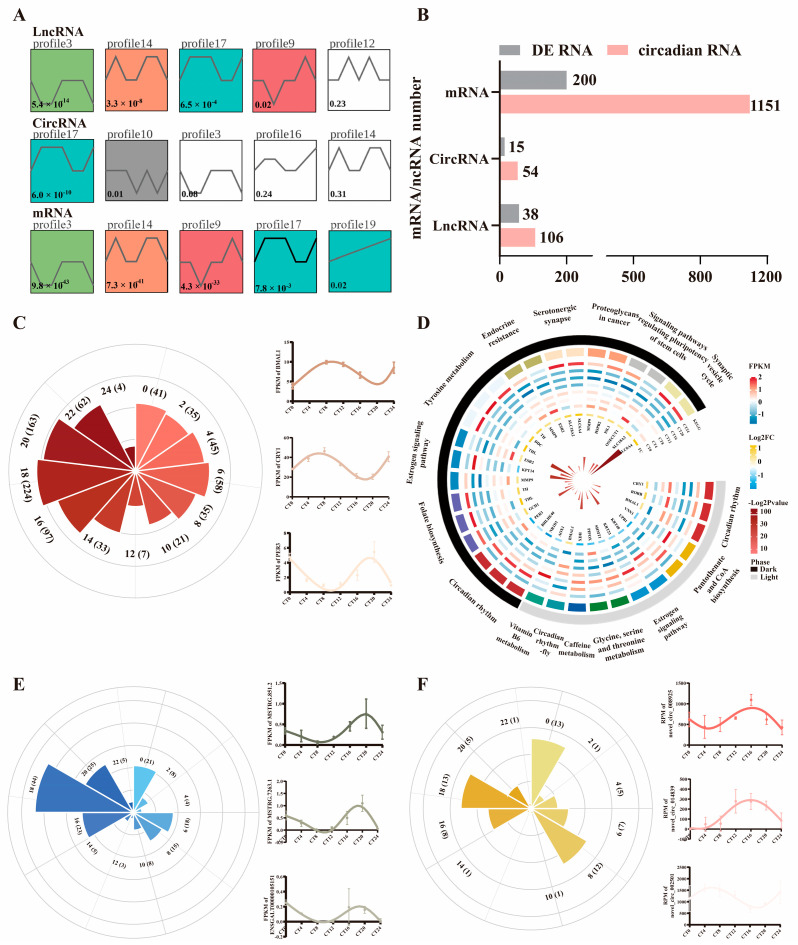
Circadian expression of differentially expressed genes and ncRNAs. (**A**) Expression trend of differentially expressed genes and ncRNAs. The colored background indicated the significant expression profiles. (**B**) Statistics for differentially expressed and circadian RNA molecules. (**C**) Distribution of time phase in one light/dark cycle for mRNA. Circadian expression of BMAL1, CRY1, and PER3 was shown in the right. (**D**) KEGG enrichment based on the phase-specific genes. Inner bars indicated the significance in log scale, the circle heatmap from inner to outer indicated the fold change, expression pattern in CT0~CT24, KEGG pathways, and phase. (**E**,**F**) Distribution of time phase in one light/dark cycle for lncRNA and circRNA, respectively. Circadian expression of partial candidate lncRNAs and circRNAs was shown in the right.

Based on the samples taken at seven time points in a 24 h cycle, we found that the transcriptional trend in profile 17 was an important expression pattern for circadian genes, lncRNAs, and circRNAs (Figure 5A). The expression patterns of circadian genes and lncRNAs were the most closely related. Through differential expression analysis, we obtained all DEGs, DElncRNAs, and DEcircRNAs of hypothalamus samples between the light and dark groups (Tables S3, S8 and S11). Next, to refine the candidate genes and lncRNAs/circRNAs associated with circadian rhythms, a rhythmic analysis of the expression at the seven CTs was conducted. A total of 1151 protein-coding genes, 106 lncRNAs, and 54 circRNAs were detected to have rhythmic significance (adjusted *p* < 0.05), including 200 DEGs, 38 DElncRNAs, and 15 DEcircRNAs (Figure 5B, Table S19). *CRY1*, *PER3*, and *BMAL1* were detected to have circadian expression, as predicted (Figure S17). For circadian DEGs, we found two phase peaks (4:00~6:00 and 16:00~22:00), and the peak was especially pronounced at 18:00 (Figure 5C). We compared the functions of the altered genes and revealed that the circadian rhythm pathway and estrogen signaling pathway functioned in both light and dark (Figure 5D). Additionally, pantothenate and CoA biosynthesis and glycine, serine, and threonine metabolism were enriched in the light, while folate biosynthesis and tyrosine metabolism were active in the dark (Figure 5D, Table S20). A similar phase pattern was drafted for circadian DElncRNAs (6:00~8:00, 16:00~20:00, and 00:00) and DEcircRNAs (4:00~8:00, 16:00~20:00, and 00:00) (Figure 5E,F).

### 2.6. Construction of a Potential lncRNA/circRNA–mRNA Network

**Figure 6:**
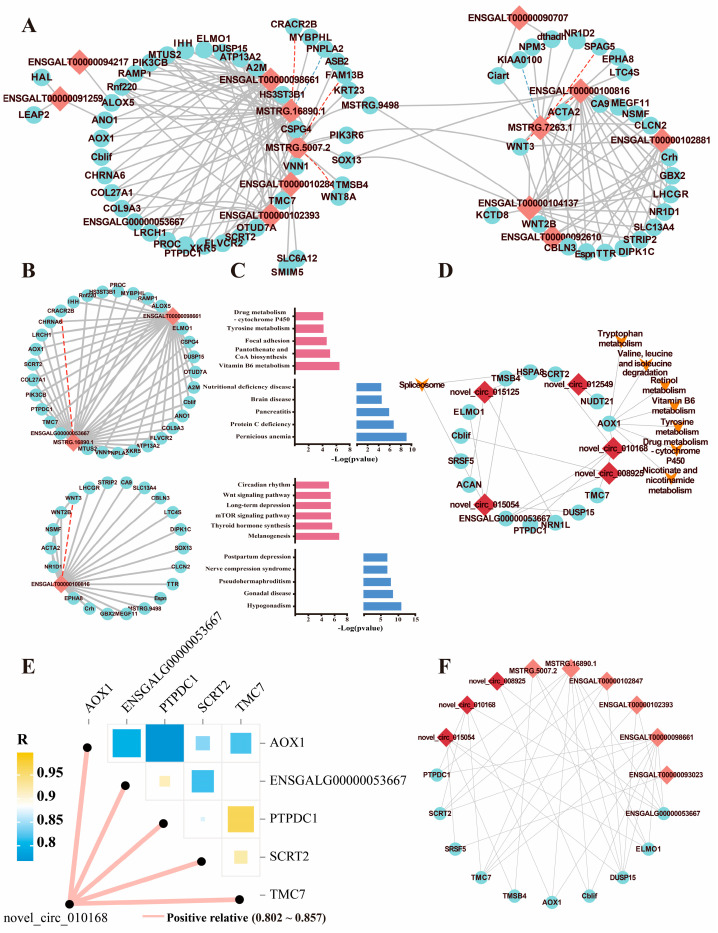
Regulatory network of lncRNA/circRNA–mRNA pairs. (**A**) Regulatory network of lncRNA-mRNA pairs. Grey lines indicated the *trans* method, red lines indicated the *cis* method, and blue line indicated the *antisense* method. (**B**,**C**) The fine network and KEGG and DO enrichment of crucial lncRNA-mRNA pairs. The upper network comprised of MSTRG.16980.1/ENSGALT00000098661-target genes, and the lower one comprised of ENSGALT00000100816–target genes. In the right, the upper rose red bars indicated the KEGG pathways, while the lower blue bars indicated the DO terms. The light red diamond indicated the lncRNAs, while the light blue circle indicated protein-coding genes. (**D**) The network of circadian circRNA-mRNA and KEGG enrichment. The dark red diamond indicated the circRNAs, the orange triangle indicated the KEGG pathways. (**E**) The relationship between novel_circ_010168 and target genes. (**F**) Regulatory network of common genes regulated by both of lncRNA/circRNA.

The competitive endogenous RNA (ceRNA) mechanism is based on the correlations between the transcript levels of various molecule pairs (e.g., lncRNA–mRNA, circRNA–mRNA) in numerous samples. In this study, only circadian mRNAs and noncoding RNAs were used to construct the molecular network. For lncRNAs, we analyzed the target genes according to the three approaches, namely, *trans*, *cis*, and *antisense* target analyses. *Trans* regulation was defined when the correlation coefficient > 0.80 and *p* < 0.001, and a total of 224 circadian lncRNA–mRNA pairs were found in *trans* relationships (Figure 6A and Figure S18, Table S21). Additionally, 33 and 2 lncRNA–mRNA pairs were identified in *cis* and *antisense* relationships, respectively (Figure S18, Table S21). MSTRG.16890.1, ENSGALT00000098661, and ENSGALT00000100816 were detected to have over 20 significant target genes (Figure 6B). A total of 25 common genes were regulated by both ENSGALT00000098661 and MSTRG.16890.1, including genes involved in vitamin B6 metabolism, tyrosine metabolism, retinol metabolism, and other functions (Figure 6B,C). ENSGALT00000100816 was proven to be correlated with melanogenesis, the Wnt signaling pathway, circadian rhythm, and other functions (Figure 6B,C). To explore the effects of promising ncRNAs on human health, we remapped the genes to the human genome and performed DO analysis. These results indicated pernicious anemia, protein C deficiency, pancreatitis, and other disorders were related to the circadian expression of MSTRG.16890.1 and ENSGALT00000098661, while gonadal and neurological diseases were influenced by ENSGALT00000100816 (Table S22). For circRNAs, only 15 circRNA–mRNA pairs were identified using Pearson correlation analysis (correlation coefficient > 0.80 and *p* < 0.001) (Figure 6D, Table S23). We found that novel_circ_010168 was associated with significantly increased expression of *AOX1*, which is involved in retinol metabolism (Figure 6D,E). The circadian genes *PTPDC1*, *SCRT2*, *TMC7*, and *ENSGALG00000053667* were also regulated by novel_circ_010168 (Figure 6E). DO analysis revealed that pernicious anemia and eye disease were associated with candidate circRNAs and target genes (Table S24). In comparing the two networks, we found that 10 common genes were significantly correlated with both lncRNAs and circRNAs, including *AOX1* and *ENSGALG00000053667* (Figure 6F).

### 2.7. Validation of Candidate lncRNAs/circRNAs and mRNAs

**Figure 7:**
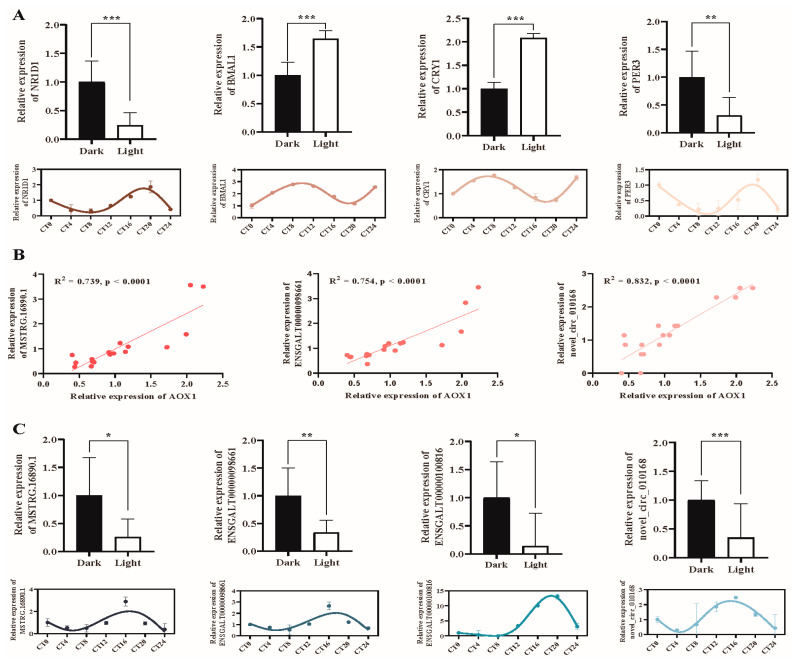
Validation of ncRNA sequencing by RT-PCR. (**A**) Expression of *NR1D1*, *BMAL1*, *CRY1*, and *PER3*. (**B**) The relationship between *AOX1* and lncRNA/circRNA. Including MSTRG.16890.1, ENSGALT00000098661, and novel_circ_010168. (**C**) Expression of MSTRG.16890.1, ENSGALT00000098661, ENSGALT00000100816, and novel_circ_010168. For (**A**,**C**), the comparison of candidate genes in light and dark was presented in the upper section, while the expression trend in seven circadian time points was presented in the lower section. * indicated *p* < 0.05, ** indicated *p* < 0.01, *** indicated *p* < 0.001.

To confirm the expression levels of the sequenced RNA molecules, we first measured the relative expression of known circadian genes (*PER3*, *CRY1*, *NR1D1*, and *BMAL1*) using RT–PCR to determine the establishment of circadian rhythm. A similar expression pattern was found between the RT–PCR and mRNA sequencing results (Figure 7A). *PER3* and *NR1D1* were expressed in the dark, while *BMAL1* and *CRY1* were highly transcribed in the light (Figure 7A). The candidate target gene *AOX1* was found to have an expression curve consistent with the sequencing result and significantly correlated with the lncRNAs and circRNAs (Figure 7B). Next, the candidate lncRNAs (MSTRG.16890.1, ENSGALT00000098661, and ENSGALT00000100816) and circRNAs (novel_circ_010168) were selected to confirm the transcriptional abundance. The three lncRNAs and novel_circ_010168 were significantly upregulated when the chickens were under light (Figure 7C). Both the results confirmed the accuracy of the sequencing results.

## 3. Discussion

Unlike mammals, birds are considered to have a central circadian system comprised of three independent endogenous circadian oscillators, the retina, pineal gland, and the SCN of the hypothalamus [25,26,27]. The retina and pineal gland are major receivers and transmitters of light to the SCN, and the SCN controls the metabolism of the pineal gland, in particularly, restricting the nocturnal synthesis of melatonin [28]. The circadian rhythm is predominantly controlled by the SCN-centered molecular network. Therefore, chickens are an ideal model for investigating the universal function and mechanism of the SCN in the hypothalamus. Zhang et al. demonstrated that the circadian rhythm of *BMAL1*, *CRY1*, etc., could be improved by applying intermittent photoperiods in the hypothalamus and proposed that the changes in photoperiod treatment might have further fed back into and strengthened the peripheral and central rhythms by activating the SCFA receptor gene pathway in the cecal wall [7]. We also confirmed that the genes *PER2*, *CRY1*, *BMAL1*, etc., were rhythmically expressed in the hypothalamus in the light/dark cycle of 24 h. It is worth noting that *CRY1* and *PER3* were expected to have a similar transcriptional pattern in SCN, but an opposite expression trend was found in the present study. The similar results have been found in chicken-related reports [6,29], but the expected results were detected in human and mice studies [30,31]. So, the difference of central circadian system of chickens and mammals could be the major cause for this issue. Additionally, monochromatic light (e.g., green and red light) and chronic corticosterone had a significant impact on the expression pattern of circadian genes in the hypothalamus of chickens [6,32]. In the current study, we provided a list of potential circadian genes; for example, *PIK3CB*, which promotes the regulation of protein kinase B and gene expression, was detected to have rhythmic expression in the hypothalamus, indicating that the functions of associated pathways such as focal adhesion and mTOR signaling pathway also operate on a light/dark cycle. It cannot be ignored that no constant light treatment was included in this study, so the pattern of circadian genes cannot be identified as the exogenous or endogenous models. Chickens were fasting in the last day, so the identified circadian genes covered the genes related to energy metabolism, etc., whose expression trend coincided with the rhythmic pattern due to the characteristics of activity. But the genes, such as *NFIL3*, *AKAP8*, and *SALL3*, highly correlated with known molecular clock genes (*CRY1*) can be identified as the candidate circadian genes.

Correlating the expression of a single gene and circadian rhythm is an inefficient method for detecting potential genes. Langfelder et al. proposed the WGCNA method to find gene modules/sets with high similarity and calculate the correlation between them and traits [33]. In this study, the light and dark were regarded as taxonomic phenotypes and submitted to the WGCNA approach. The genes of the dark green module (e.g., *PER3*, *BMAL1*, and *CRY1*) were highly correlated with circadian rhythm, and the circadian rhythm of the expression of these genes was also confirmed in the pineal gland and retina of chickens [34]. Additionally, in the chicken epiphysis, the activity of heat shock factor 1 is triggered by light, and the modification of heat shock factor 1 shows a circadian rhythm [35,36,37]. We also found that members from the HSP gene family in the dark green module (e.g., *HSPA5*, *HSPA8*, and *HSP40*) changed with the light/dark cycle, which may have been driven by the alteration of temperature. Retinol (vitamin A) is important to the circadian timing system, and its deficiency disrupts circadian gene expression [38,39]. We found that the retinol metabolism-associated genes *ADH6*, *ALDH1A1*, *CYP1A1*, and others were differentially expressed in the light and dark periods in the hypothalamus, and these genes were mainly transcribed after 16:00. These findings may provide novel insights into the molecular link between nutritional regulation and the circadian system. Glycolysis/gluconeogenesis was more active at night due to the higher expression of *ALDOB*, *ALDH*, *G6PC1*, etc., which was demonstrated by Isobe et al.’s report [40].

Studies have revealed hundreds of lncRNAs that are expressed rhythmically in different tissues, implying their functions in the circadian clock system [23,41,42,43]. LncRNAs are characterized by tissue specificity and weak conservation, and more than 11% of novel lncRNAs have been assembled. Here, 38 source lncRNAs with differential and circadian expression in the light/dark cycle were used to construct the regulatory network. Three core lncRNAs were selected, and target genes *AOX1*, *PIK3CB*, and *WNT2B* were found to have potential functions in circadian rhythm by amino acid metabolism (e.g., tyrosine, and retinol) and the Wnt signaling pathway, among others. The expression of the gene *AOX1* has been reported to be correlated with reproductive performance and follicular development, as well as osteogenic differentiation [44,45], and it plays an important role in retinol metabolism. A study on monarch butterflies indicated that retinol in the brain rather than the compound eye functioned in photoperiod responsiveness [46]. Yoshida et al. demonstrated that serum retinol enhanced the expression of *CLOCK*/*BMAL1* genes [47]. Additionally, *AOX1* was involved in hepatic metabolism and heat production, which may be related to the activity characteristics within 24 h [48,49]. In total, the current study inferred that *AOX1* contributed to the regulation of the light/dark cycle in the hypothalamus and was controlled by MSTRG.16890.1 and ENSGALT00000098661 in chickens. Dysfunction of circadian rhythm was highly associated with hyperactivation of the Wnt signaling pathway in human disease cases (e.g., autism spectrum disorder, colorectal cancer, and osteoarthritis) [50,51,52], suggesting a higher risk of disease gene expression in the dark according to the phase distribution of *WNT2B* gene expression (peak at 20:00) in the current study.

CircRNAs, consisting of one or more exons, have conserved biogenesis and are widely expressed in different species and tissues, especially in the brain and synaptic terminals [14,53,54,55]. These observations indicate the potential of circRNAs in the regulation of circadian rhythm. Studies have demonstrated that circRNAs influence melatonin metabolism and circadian rhythm by sponging miRNAs in the pineal gland [20,56]. CircRNA Cdr1as strongly regulates light entrainment during a 12:12 h light/dark cycle in the SCN tissue [21]. Here, we identified five circRNAs that are differentially expressed in light and dark conditions, and a network covering 15 circadian circRNA-mRNA pairs was constructed in accordance with the rhythmic pattern. This confirmed the significance of *AOX1* and *HSPA8*, regulated by novel_circ_010168 and novel_circ_015125, respectively, in the circadian rhythm mechanism. Additionally, in this network, *SRSF5* has been proven to play a role in circadian rhythm [57], and loss of *BMAL1* decreased the expression of the matrix-related gene *ACAN* [58], indicating the potential impacts of novel_circ_015125 and novel_circ_015054 on circadian rhythm in the SCN. Additionally, opposing dynamics of lncRNAs and circRNAs were found during one light/dark cycle, indicating that the investigated lncRNAs and circRNAs coordinate their effects on circadian rhythm by influencing different pathways, e.g., mTOR signaling pathway and protein processing in the endoplasmic reticulum. Okazaki et al. proved that circadian oscillation of mTOR activity is regulated by central clock systems [59], and the function of HSP gene family members in protein processing in the endoplasmic reticulum was also demonstrated in a study by Hatori et al. study [37]. In total, we have comprehensively characterized expression pattern of lncRNAs and circRNAs in one light/dark cycle and refined the circadian ncRNAs–mRNAs networks, providing novel insights into the establishment mechanisms and biomarkers of circadian rhythm.

## 4. Materials and Methods

### 4.1. Animals

A total of 105 newly hatched chickens (Hy-Line chicken, Jiande Jianke Breeding Co., Ltd., Hangzhou, China) were randomly reared in 7 circadian groups (3 replicates in each group and 5 individuals in each replicate) under white light (400–700 nm). All animals were kept under 15 ± 0.3 lx at the level of the chicken’s head on a 12:12 h light/dark cycle in the 8th week as reported previously [6]. Chickens had free access to feed and water. The dietary composition and nutritional content at all stages are shown in Table S25. The humidity was controlled at 50~60%. The room temperature was maintained at 33 ± 1 °C in the first week and decreased by 2~3 °C every week until 21 °C was reached in the 6th week.

### 4.2. Sample Collection

On day 56, chickens were sacrificed every 4 h at the following time points: CT0, CT4, CT8, CT12, CT16, CT20, and CT24 (CT, circadian time). One chicken selected randomly from each replicate in every group/circadian time point was slaughtered. The hypothalamus tissue was rapidly dissected out and instantly frozen using liquid nitrogen and then stored at −80 °C.

### 4.3. Total RNA Isolation and Quality Control

Total RNA was extracted using the Trizol reagent kit (Invitrogen, Carlsbad, CA, USA) according to the manufacturer’s protocol. RNA quality, including concentration (115~297 ng/μL) and RIN (9.2~9.7), was assessed using an Agilent 4200 Bioanalyzer (Agilent Technologies, Palo Alto, CA, USA) and checked using RNase-free agarose gel electrophoresis. After total RNA was extracted, rRNAs were removed using probe hybridization and incubation with RNase H and DNase I to retain mRNAs and ncRNAs.

### 4.4. Library Preparation and Sequencing of Noncoding RNAs

The enriched mRNAs and ncRNAs were broken into fragments and reverse transcribed into cDNA with random primers. Then, the cDNA was purified using a QIAquick PCR extraction kit (Qiagen, Venlo, The Netherlands) and end paired, and poly(A) and universal adapters were added. After cDNA digestion using uracil-N-glycosylase, the products were size selected by agarose gel electrophoresis, PCR amplified, and sequenced using the NovaSeq 6000 platform. Four libraries were removed due to the low quality. More than 60 million reads per sample were generated.

### 4.5. Quality Control, rRNA Mapping and Removal, and Genome Alignment

The raw data were first trimmed and filtered by fastp software [60], and reads exceeding any of the following thresholds were removed: (1) adapter sequences; (2) >10% of unknown nucleotides (N); and (3) >50% of low-quality (Q score < 20) bases. Additionally, Bowtie2 was used to map reads to the ribosome RNA (rRNA) database. Any rRNA mapped reads were then removed. Then, the qualified reads were aligned to the chicken reference genome (GRCg6a, http://ftp.ensembl.org/pub/release-106/fasta/gallus_gallus/dna/, accessed on 10 August 2023) using HISAT2 v2.2.1 software (-rna-strandness RF) [61]. The SAM files were next converted, sorted, and indexed using Samtools v1.12 software [62]. For mRNA, the transcripts were assembled with referrence to the chicken genome GTF file (http://ftp.ensembl.org/pub/release-106/gtf/gallus_gallus/, accessed on 10 August 2023) using StringTie v2.1.6 software [63], and the assembled transcripts of each sample were merged using the ‘--merge’ parameter. After merging, the aligned results were merged again according to the merged GTF file. The final assembled results were used for gene quantification using a prepDE.py script (−l 150).

### 4.6. Prediction and Quantification of Novel lncRNAs

The transcripts were assembled as described above using StringTie v2.1.6 software [63]. Three software programs, CNCI [64], CPC2 [65], and Feelnc [66], were used to predict the protein-coding ability of novel transcripts with default parameters. Transcripts predicted as non-protein-coding by all three programs were regarded as novel lncRNAs. We obtained the known lncRNAs by mapping the results and annotated the lncRNAs based on the chicken genome GTF file (http://ftp.ensembl.org/pub/release-106/gtf/gallus_gallus/, accessed on 10 August 2024). According to their locations in the genome, lncRNAs were divided into five groups: intergenic, bidirectional, intronic, antisense, and sense lncRNAs.

For each lncRNA transcript, the fragment per kilobase of transcript per million mapped reads (FPKM) value was calculated to quantify the transcription abundance. The use of FPKM values is conducive to eliminating the effect of transcript length and sequencing data amount on expression level calculations.

### 4.7. Identification and Quantification of circRNAs

CircRNAs were identified after alignment using CIRIquant software; the details of this process were as described in a previous report [67,68]. CircRNAs were typed according to the location and exon type as annotated_exons, antisense, exon_intron, intergenic, intronic, or one_exon. The distribution on chromosomes and length distribution were summarized.

The reads per million mapped reads (RPM) values were used to scale the back-spliced junction reads and quantify the circRNA abundance. This method contributes to eliminating the effect of sequencing data amount on the calculation of expression level.

### 4.8. Weighted Gene Co-Expression Network Analysis

To identify genes under the control of circadian rhythms, a co-expression network based on the transcriptional abundance of mRNAs from the hypothalamus was constructed using the weighted gene co-expression network analysis (WGCNA) method [33]. We first established an expression matrix of 16,342 qualified genes at seven time points. The soft threshold (β = 6) was determined by the scale-free distribution result (R^2^ > 0.9) (Figure S19). Next, step-by-step and dynamic cutting methods were applied to construct a co-expression network and detect gene modules with the parameters: minModuleSize = 50 and mergeCutHeight = 0.25 (Figure S20). A correlation coefficient > 0.5 was set as the threshold for the circadian gene modules. Within the circadian gene modules, the genes with |KME| > 0.8 were defined as the hub genes.

### 4.9. Detection of Differentially Expressed Genes/Noncoding RNAs

The gene expression levels of protein-coding genes and ncRNAs were normalized by DESeq2 [69], and differentially expressed genes (DEGs) or ncRNAs (DElncRNAs and DEcircRNAs) with a fold change >1.5 or <0.67 and *p* value <0.05 between light and dark groups were identified based on raw transcript count.

### 4.10. Prediction of Periodicity of lncRNAs, circRNAs, and mRNAs

The rhythmicity of protein-coding genes, lncRNAs, and circRNAs was assessed using the *meta2d* function in the MetaCycle package [70], and the JTK-cycle method was applied. Genes and ncRNAs were considered to have circadian expression when the adjusted *p* value was <0.05. The phases in which each gene and ncRNA were expressed were counted.

### 4.11. Gene Trend Analysis for lncRNAs, circRNAs, and mRNAs

Short Time-series Expression Miner (STEM) analysis was performed to cluster differentially expressed circadian genes and ncRNAs with the same time trend [71]. An adjusted *p* value of less than 0.05 was considered to indicate significant clustering, and the genes or ncRNAs clustered within the same group showed the similar expression patterns.

### 4.12. Construction of the ceRNA Network for lncRNAs/circRNA–mRNAs Pairs

The lncRNA–mRNA and circRNA–mRNA networks were developed based on the potential functional relationships among them. Based on the ceRNA theory, lncRNAs can sponge miRNAs to influence mRNA abundance. For lncRNA, three regulatory methods (*trans*, *cis*, and *antisense*) were considered. Pearson correlation analysis was conducted to detect the target genes in *trans*. The significant LncRNAs–mRNAs pairs were defined as those with a coefficient of > 0.8 and a *p* value <0.0001. Genes located on the same strand within 10 kb of lncRNAs were considered *cis* target genes, while those on the opposite strand were regarded as *antisense* target genes. For CircRNA, target genes were detected using Pearson correlation analysis and the same threshold as lncRNAs. The networks were visualized by Cytoscape 3.10 [72].

### 4.13. Functional Enrichment Analysis

To explore the potential biological functions and pathways involving candidate DEGs, ncRNA target genes, and genes enriched in specific time phases, Kyoto encyclopedia of genes and genomes (KEGG), Reactome, and disease ontology (DO) enrichment analyses were performed and visualized using the online tool OmicShare (https://www.omicshare.com/, accessed on 24 October 2024).

### 4.14. Validation of Candidate lncRNAs, circRNAs, and mRNAs

To verify the mRNA and ncRNA sequencing results, we performed real-time PCR (RT–PCR) to determine the relative expression of selected mRNAs (*BMAL1*, *PER3*, *CRY1*, *NR1D1*, *AOX1*) and ncRNAs (MSTRG.16890.1, ENSGALT00000098661, ENSGALT00000100816, novel_circ_010168). The gene-specific primers were designed using Oligo 6.0 software (Table 1).

### 4.15. Statistical Analysis

Comparisons of the expression of novel and known lncRNAs and mRNAs were conducted using the Wilcoxon signed-rank sum test, and the expression of candidate genes and ncRNAs between light and dark group were compared using student’s *t* test and Pearson correlation analysis, all the statistics were conducted in SPSS 25.0 software. A *p* value < 0.05 was considered to indicate statistical significance.

## 5. Conclusions

We generated a rhythmic expression profile of lncRNAs, circRNAs, and mRNAs across seven time points (CTs) in one light/dark cycle. Refined ncRNAs–mRNAs networks have been constructed to explore the mechanisms and biomarkers of circadian rhythm. We found three lncRNAs (MSTRG.16890.1, ENSGALT00000098661, and ENSGALT00000100816) and one circRNA (novel_circ_010168) as predominant regulatory molecules, influencing *AOX1*, *PIK3CB*, *WNT3*, and other genes in different pathways and time phases.

## Figures and Tables

**Table 1:** Primers for candidate genes and ncRNAs in RT-PCR.

ID	Source Gene	Primer	Product Size
*PER3*	/	F: 5′-AGAGCTTTCGTTGGTACAGCC-3′	188
		R: 5′-ACCCAGTTTTCAGTCGCTCA-3′	
*BMAL1*	/	F: 5′-GAAAGTAGGTCAGGGACGGG-3′	287
		R: 5′-TGTCCATCCTTTGGTCTGCC-3′	
*CRY1*	/	F: 5′-TGGCGGTTTCTGCTTCAGT-3′	219
		R: 5′-GACCTCCACTCCAGCTTCAC-3′	
*NR1D1*	/	F: 5′-AGTGCGGTGGAGGCGTTC-3′	254
		R: 5′-CAGCACATCTCGTTGCCCG-3′	
*AOX1*	/	F: 5′-AAATGCCGATCCCGAACAGA-3′	316
		R: 5′-GGACATCACCATTCCAGGGG-3′	
*MSTRG.16890.1*	*PNPLA2*	F: 5′-GCGCCCGTACCTATCTTTCA-3′	491
		R: 5′-GCTGCCGCCTCTTAACTCC-3′	
*ENSGALT00000098661*	*ENSGALG00000039896*	F: 5′-TGGACTTGTGTGCCTGGAAG-3′	379
		R: 5′-GTGGTGGATTGCCCTACTGT-3′	
*ENSGALT00000100816*	*ENSGALG00000031974*	F: 5′-TTCCTCAGTACCAGTGGGGT-3′	331
		R: 5′-AGGAGCCCCGTTGACTAAGA-3′	
*novel_circ_010168*	*CREB5*	F: 5′-GAACTTGGAGCAGGAGAGGC-3′	173
		R: 5′-GAAAGCGTGTCGGTGTAGGA-3′	
*GAPDH*	/	F: 5′-CGTCTGGAGAAACCAGCCAA-3′	315
		F: 5′-AACAAAGGGTCCTGCTTCCC-3′	
*ACTB*	/	F: 5′-GAGAAATTGTGCGTGACATCA-3′	152
		R: 5′-CCTGAACCTCTCATTGCCA-3′	

## Data Availability

The raw data of noncoding RNA generated from this study were deposited to the GSA database (accession number: CRA013579). The gtf and fasta files of noncoding RNAs were presented in Supplementary Information files. All the data produced in this study can be available from the authors upon academic request.

## Supplementary Materials

The following supporting information can be downloaded at: https://www.mdpi.com/article/10.3390/ijms25021161/s1.
