# Supplementary figures and images for: Novel Insights into the Circadian Rhythms Based on Long Noncoding and Circular RNA Profiling
Source: Int J Mol Sci. 2024 Jan 18;25(2):1161. doi: 10.3390/ijms25021161 (PMC10816401; doi:10.3390/ijms25021161)

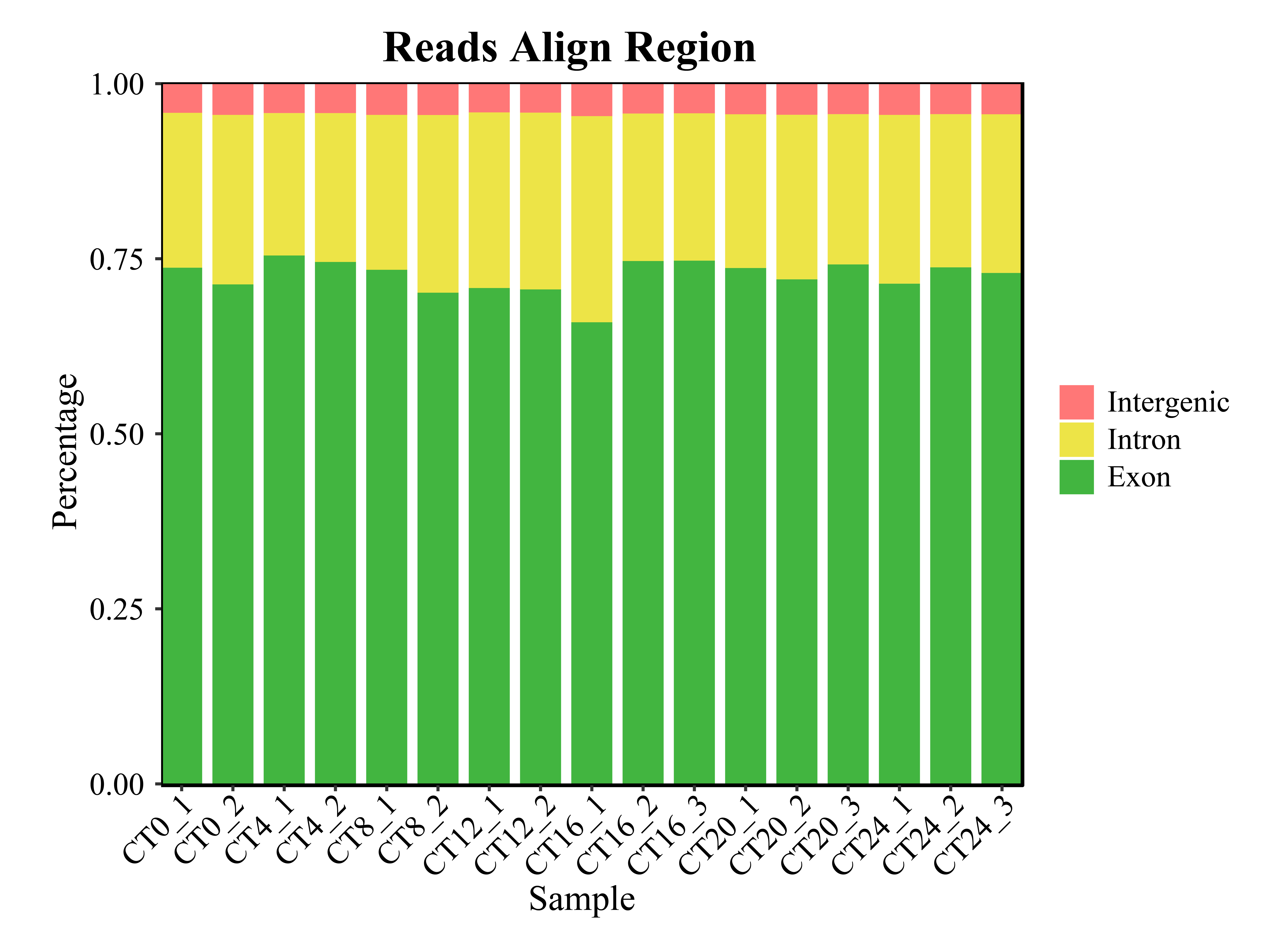

Supplement: Supplementary file 1 [file ijms-25-01161-s001.zip › Figure S1.jpg]

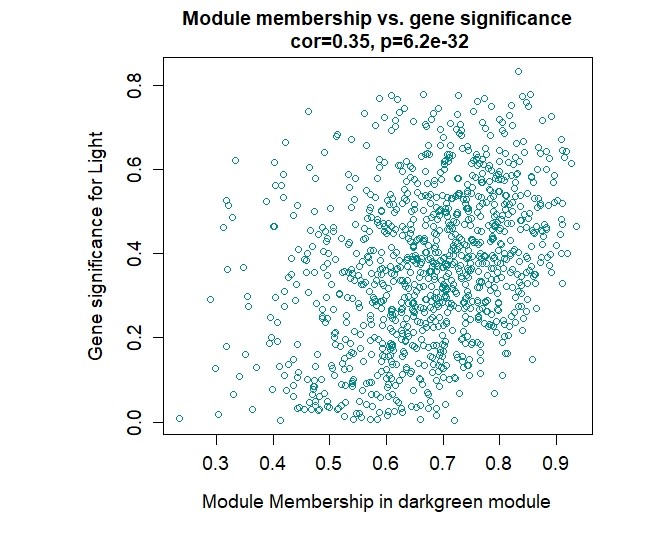

Supplement: Supplementary file 1 [file ijms-25-01161-s001.zip › Figure S12.jpg]

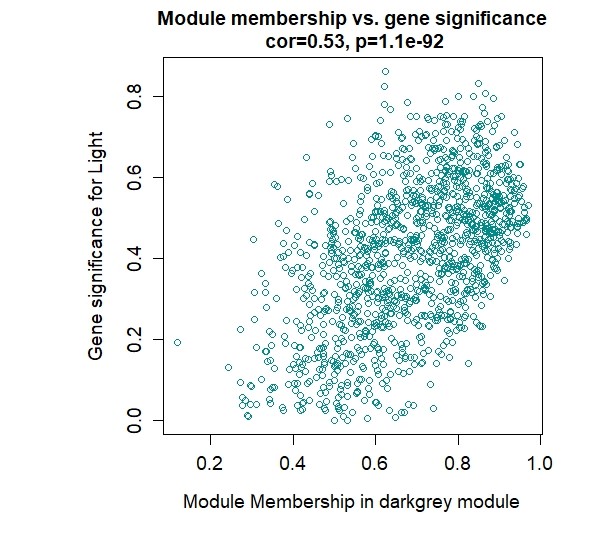

Supplement: Supplementary file 1 [file ijms-25-01161-s001.zip › Figure S13.jpg]

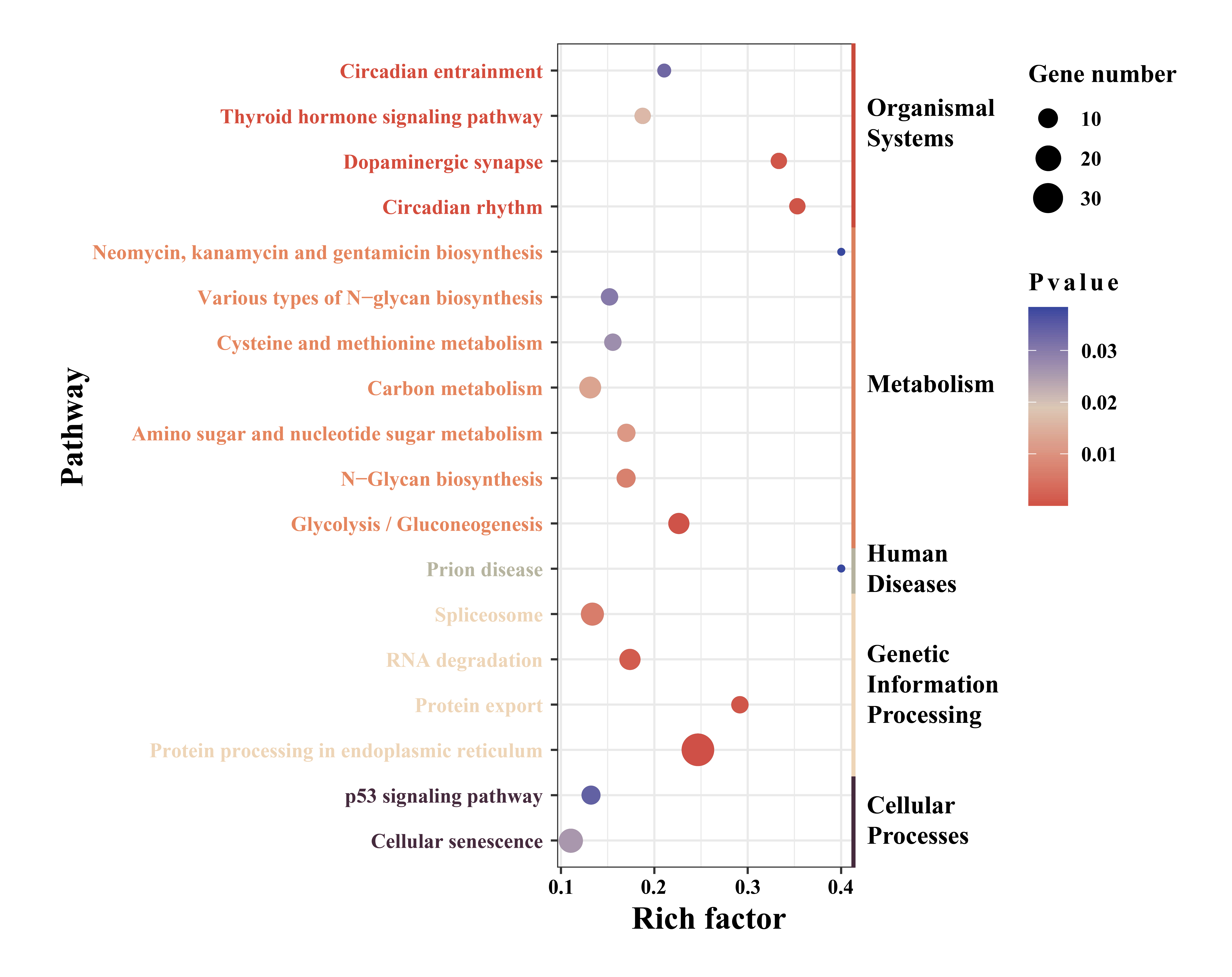

Supplement: Supplementary file 1 [file ijms-25-01161-s001.zip › Figure S14.jpg]

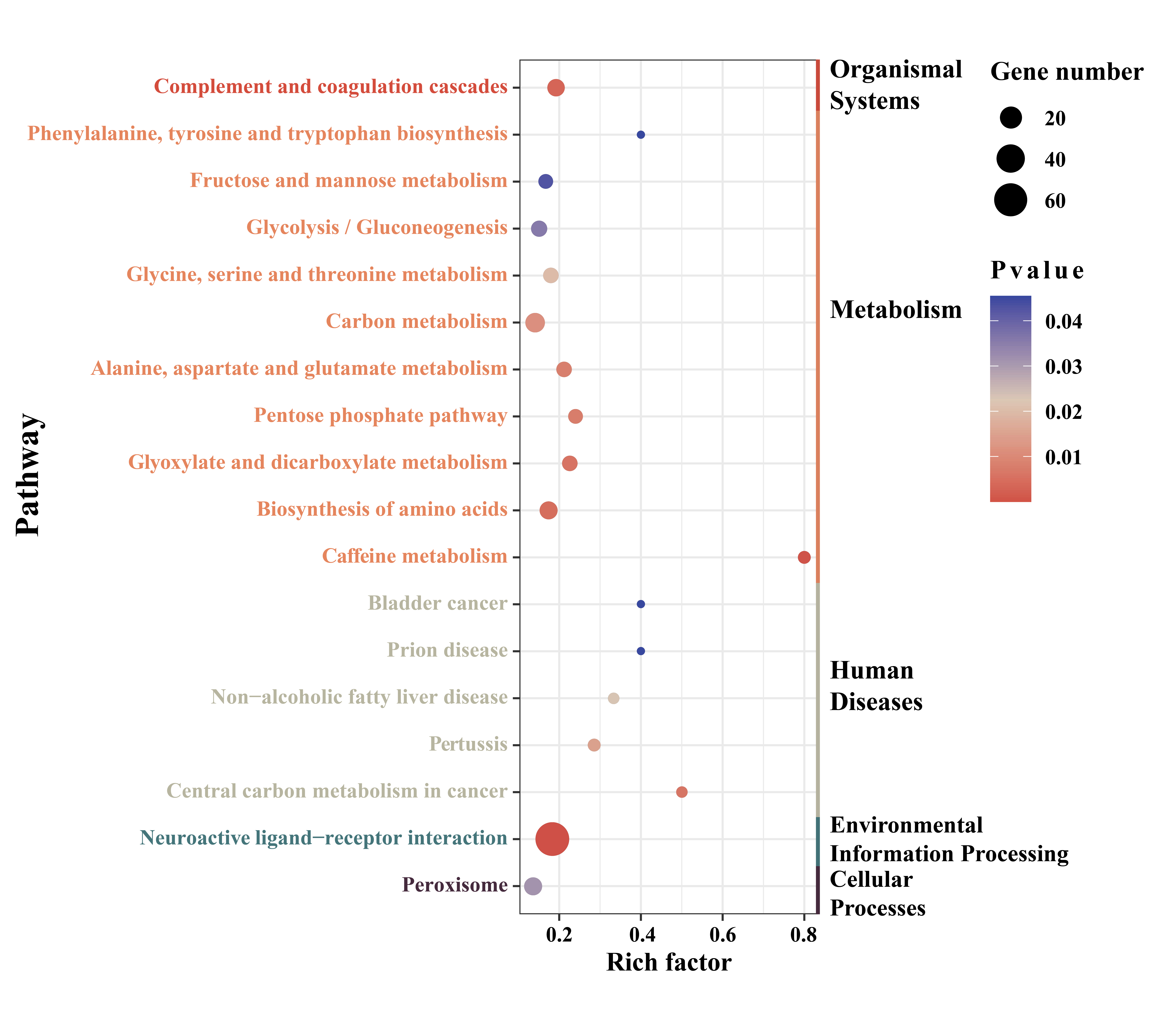

Supplement: Supplementary file 1 [file ijms-25-01161-s001.zip › Figure S15.jpg]

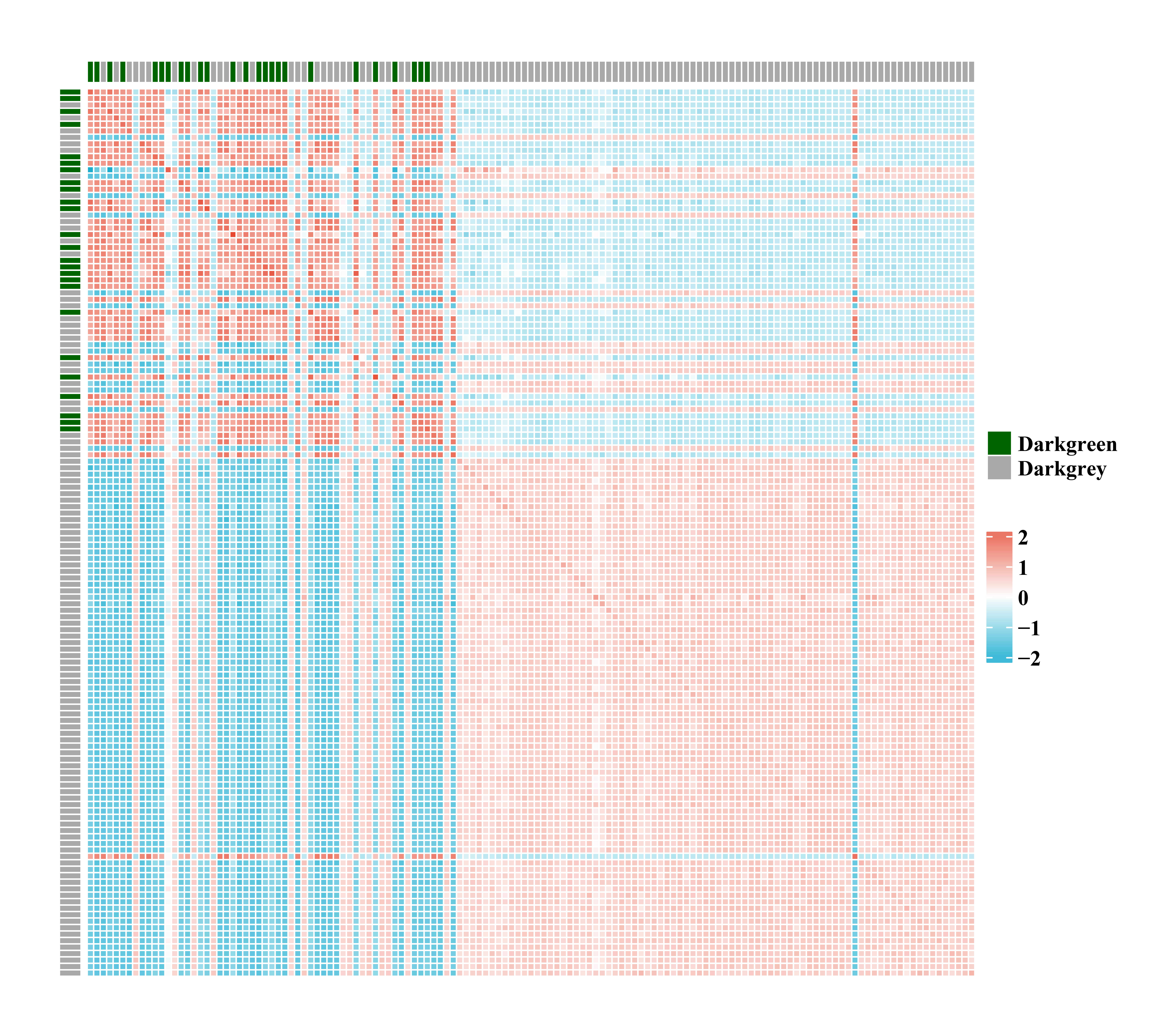

Supplement: Supplementary file 1 [file ijms-25-01161-s001.zip › Figure S16.jpg]

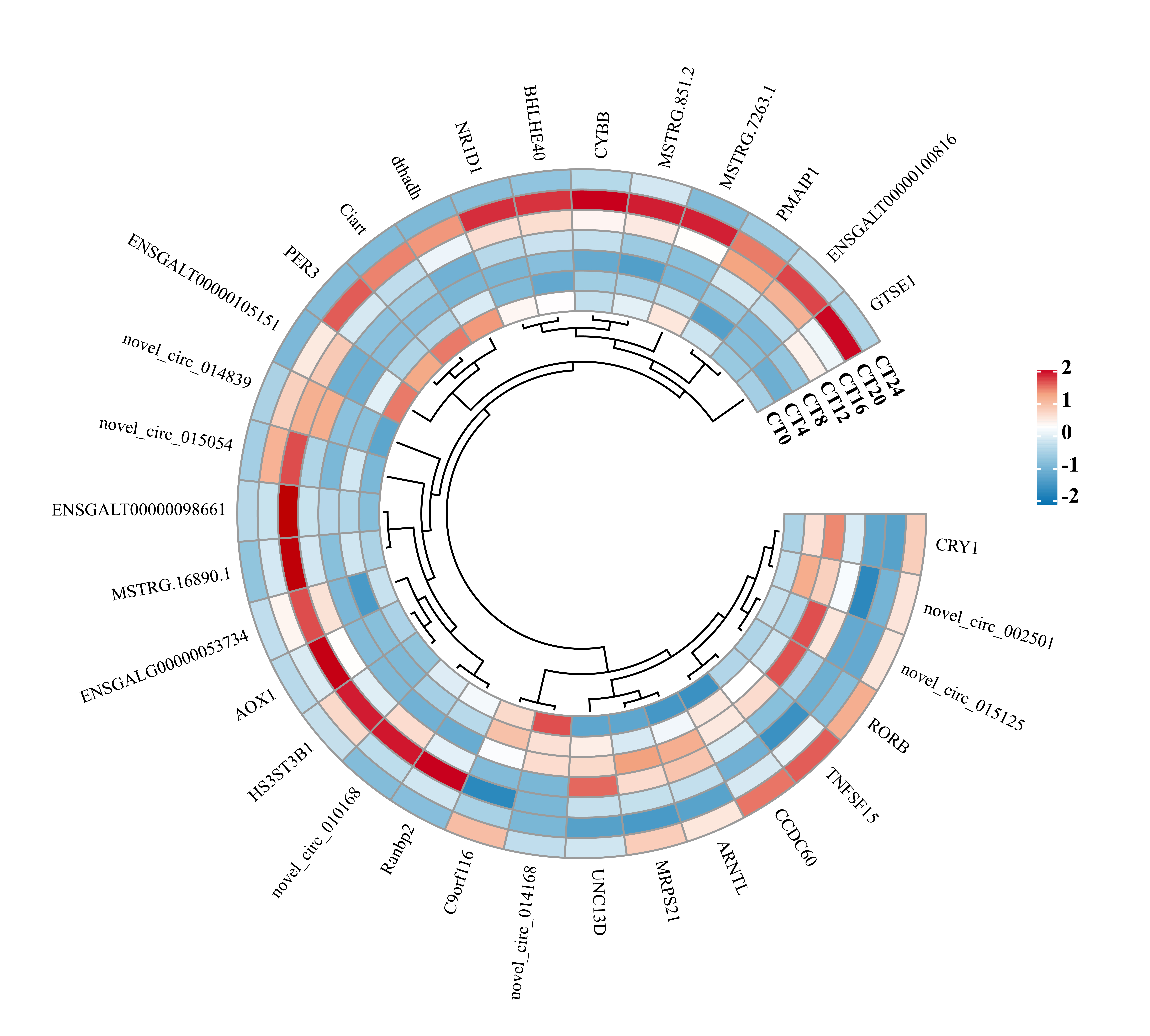

Supplement: Supplementary file 1 [file ijms-25-01161-s001.zip › Figure S17.jpg]

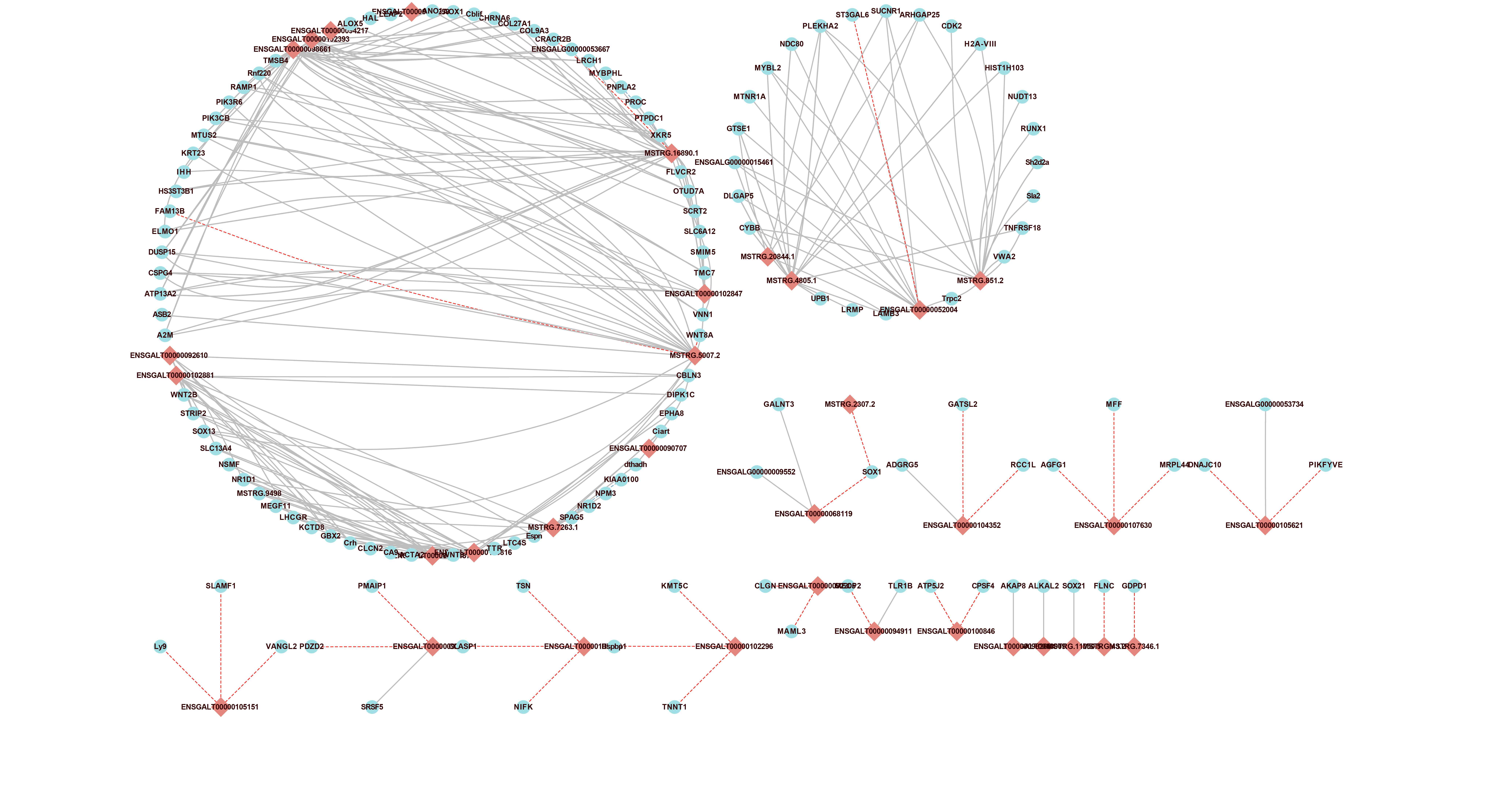

Supplement: Supplementary file 1 [file ijms-25-01161-s001.zip › Figure S18.jpg]

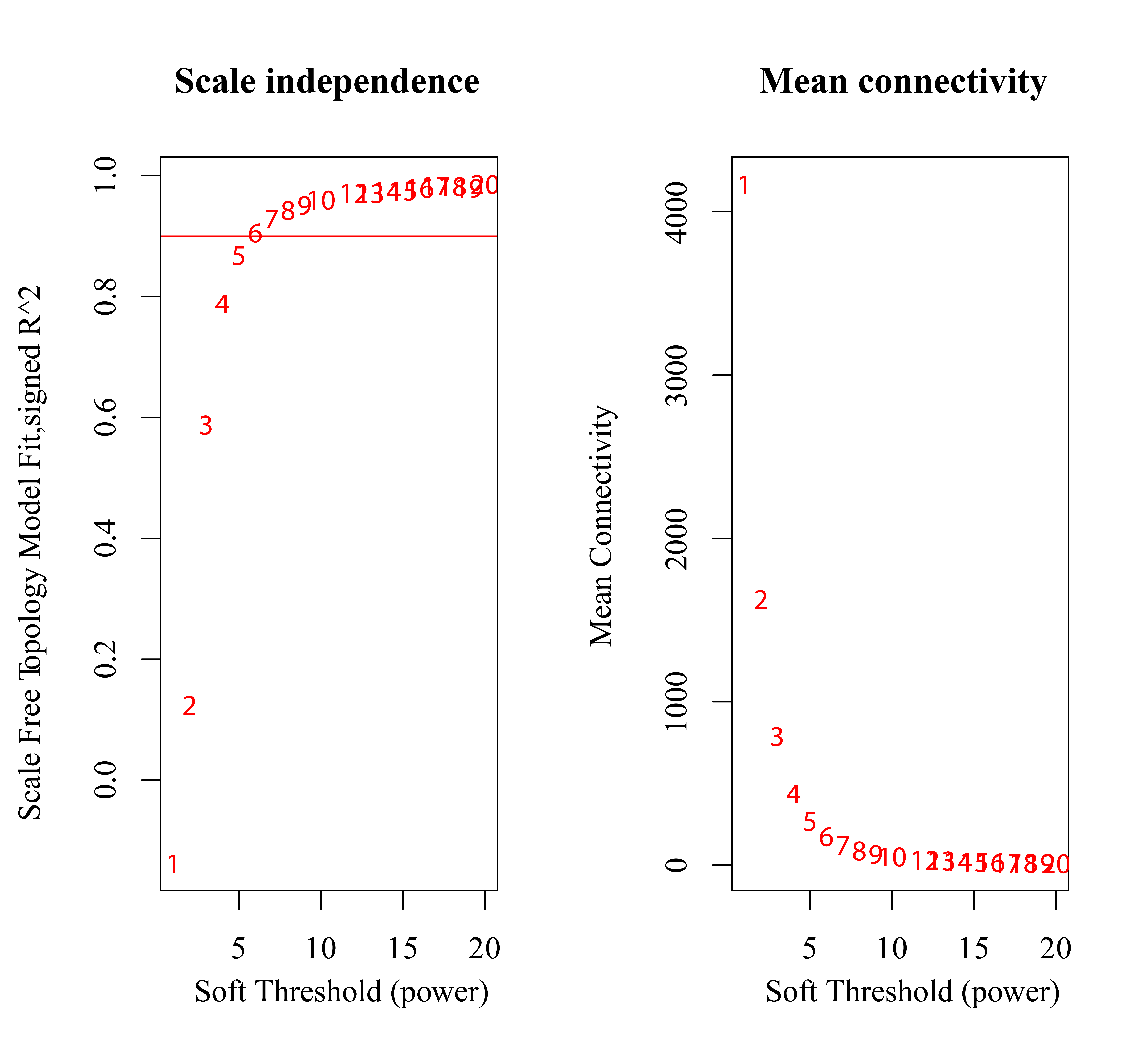

Supplement: Supplementary file 1 [file ijms-25-01161-s001.zip › Figure S19.jpg]

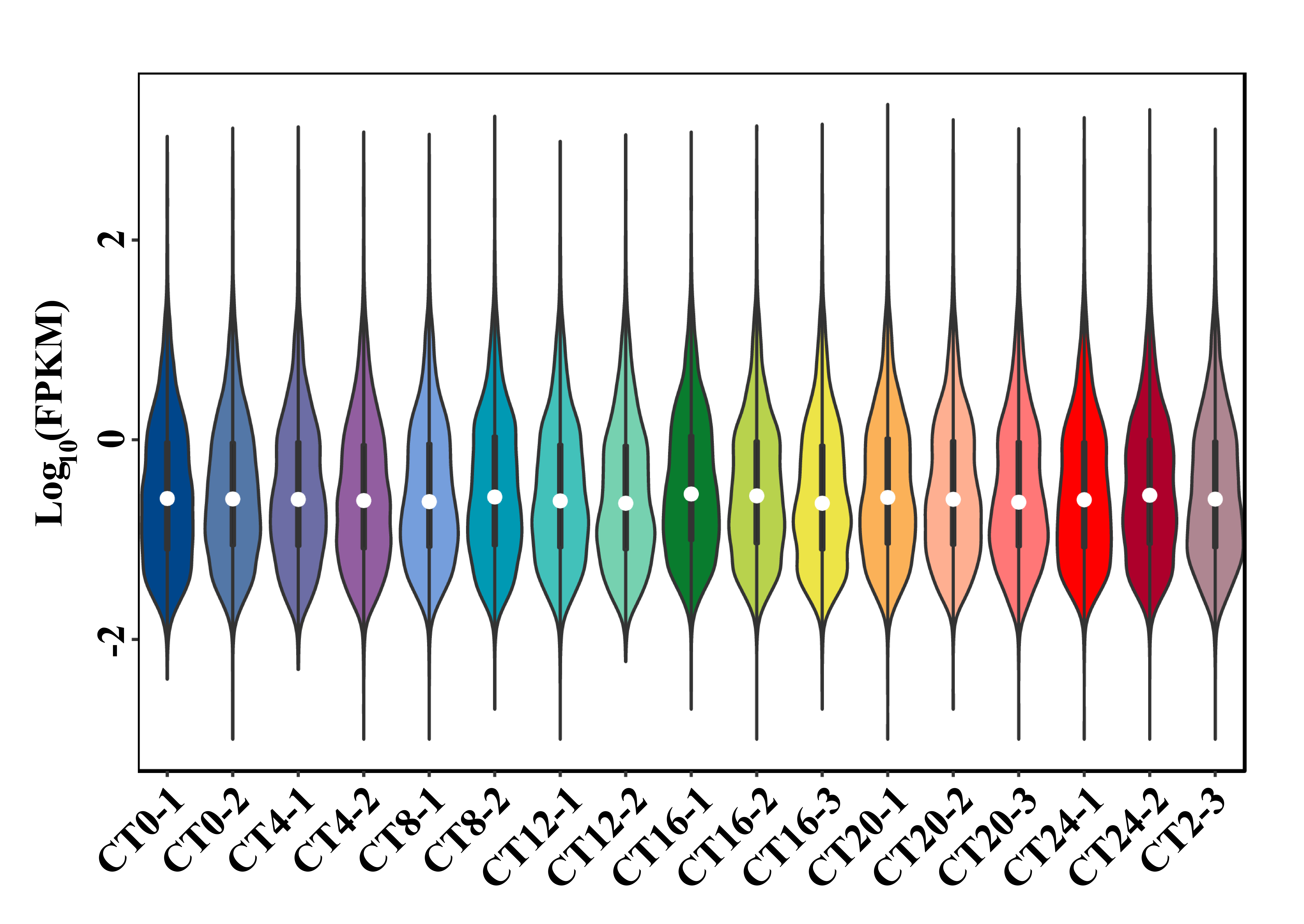

Supplement: Supplementary file 1 [file ijms-25-01161-s001.zip › Figure S2.jpg]

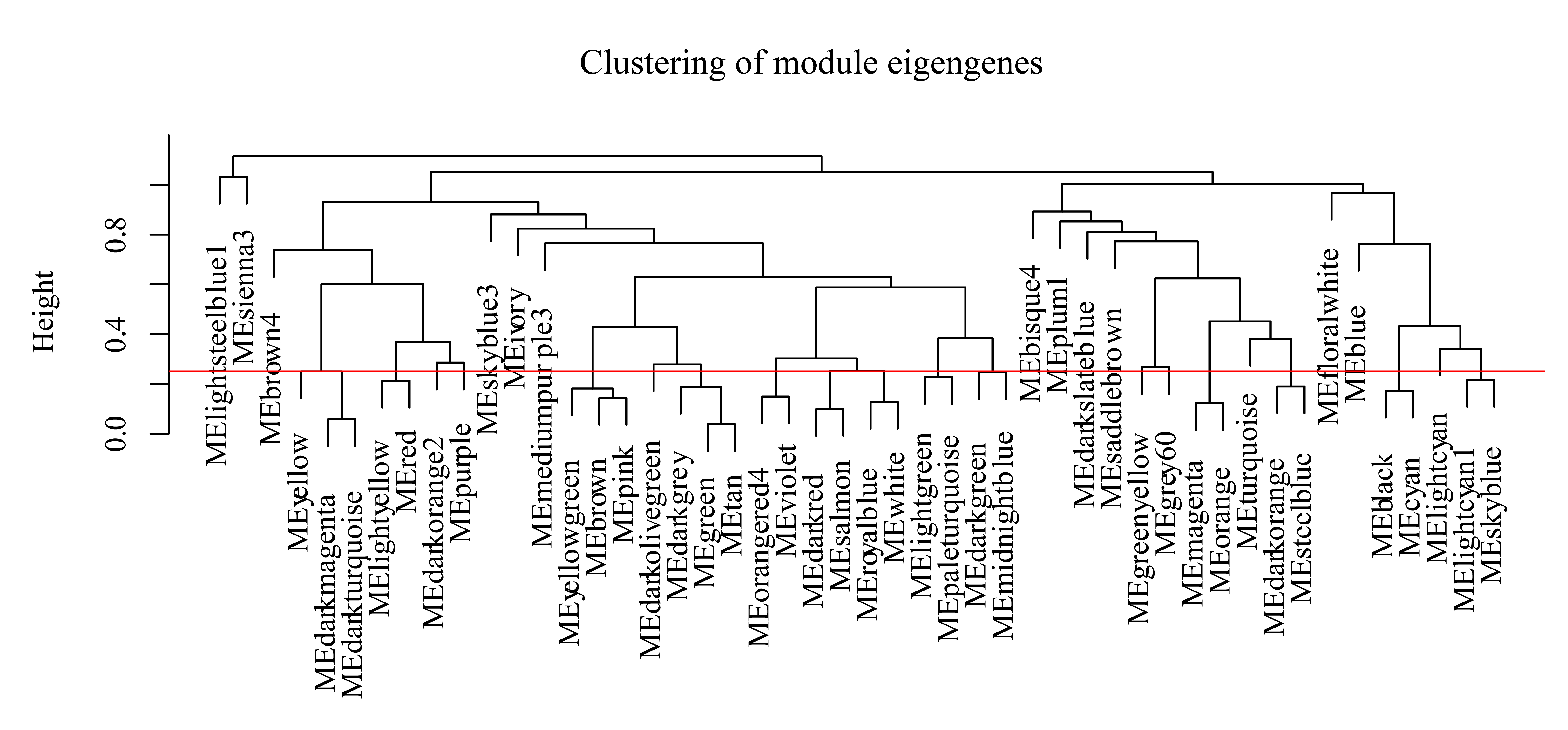

Supplement: Supplementary file 1 [file ijms-25-01161-s001.zip › Figure S20.jpg]

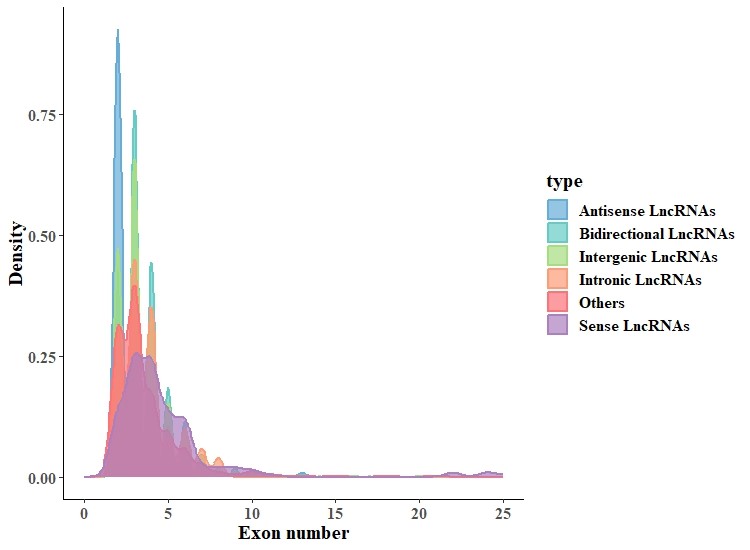

Supplement: Supplementary file 1 [file ijms-25-01161-s001.zip › Figure S3.jpg]

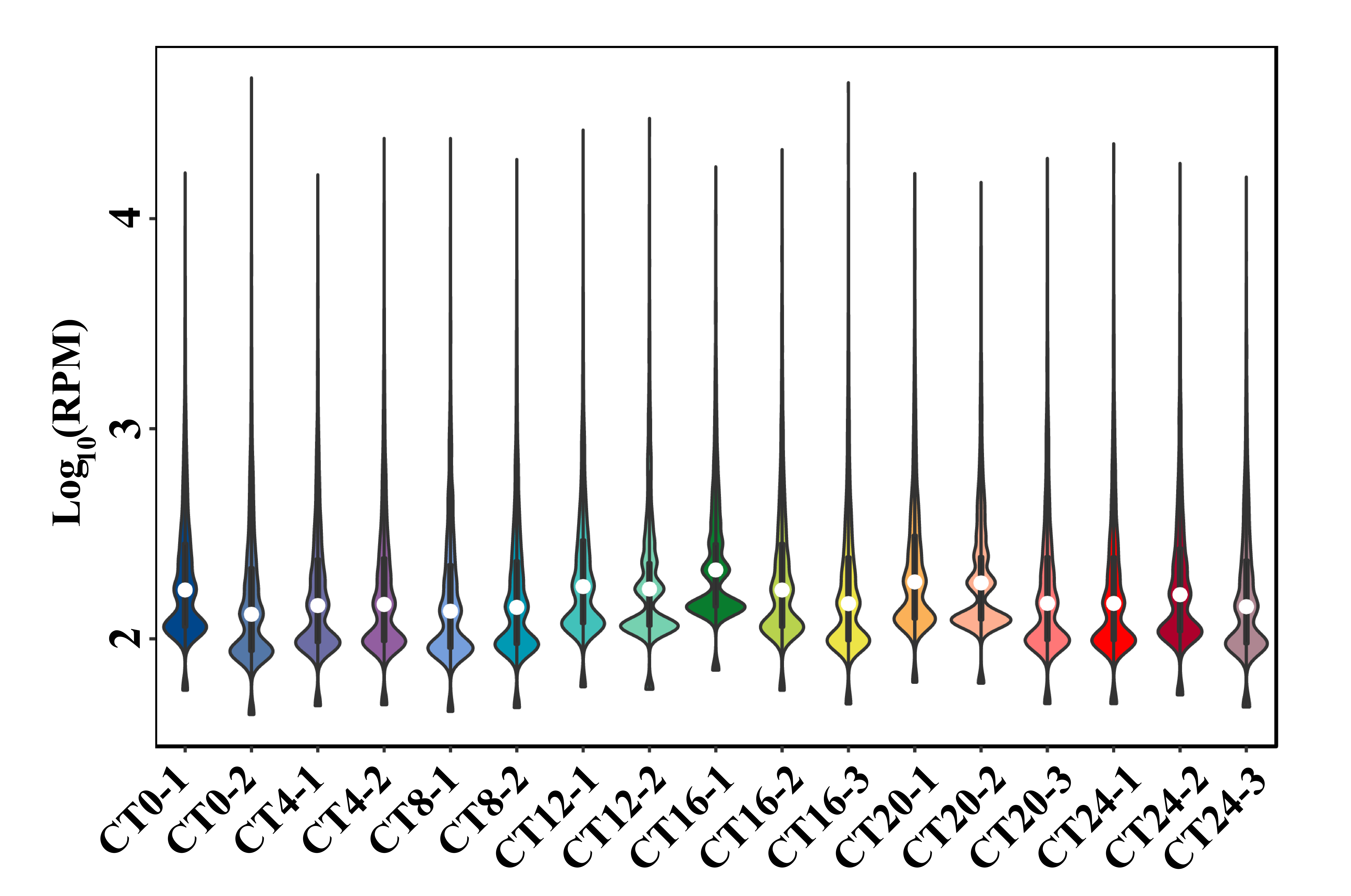

Supplement: Supplementary file 1 [file ijms-25-01161-s001.zip › Figure S4.jpg]

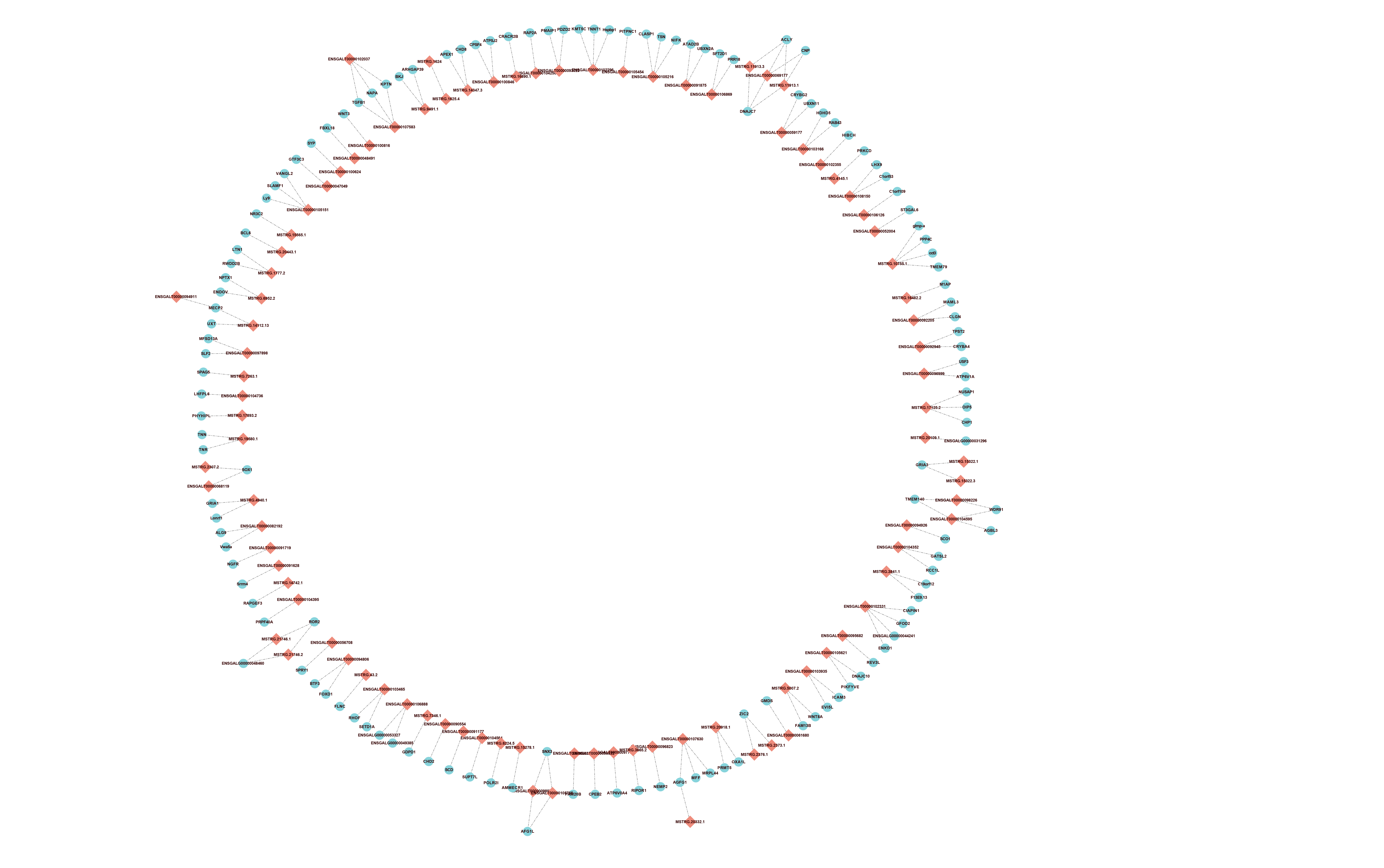

Supplement: Supplementary file 1 [file ijms-25-01161-s001.zip › Figure S7.jpg]
